# Multiple Adverse Outcomes of Intrauterine Devices in One Patient: A Case Report

**DOI:** 10.7759/cureus.47868

**Published:** 2023-10-28

**Authors:** Ariel Hall, Reetu Grewal

**Affiliations:** 1 College of Medicine, University of Florida, Gainesville, USA; 2 Department of Family and Community Medicine, University of Florida College of Medicine – Jacksonville, Jacksonville, USA

**Keywords:** multiple adverse outcomes, copper intrauterine contraceptive devices, primary care, adverse outcomes, intrauterine devices

## Abstract

Intrauterine devices (IUDs) are commonly used, effective forms of long-acting removable contraceptives that may be inserted by primary care providers. Adverse outcomes with copper IUDs specifically have been extensively documented; however, there is little guidance on whether to offer an IUD to a patient who has already experienced adverse outcomes related to IUDs. In this case report, our patient experienced three complications with three different copper IUDs, including a spontaneous expulsion, a fragmented device, and a retained device in addition to two unintended pregnancies. In our view, a different form of contraception should be offered for a patient that has already experienced multiple adverse outcomes related to IUDs.

## Introduction

Intrauterine devices (IUDs) are one of the most commonly used forms of long-acting removable contraceptives [1]. There are both hormonal and copper formulations of IUDs, and the commercially available option for copper IUDs in the United States is Paragard. Patients can have IUDs inserted and removed in the outpatient setting by any appropriately trained primary care physician or gynecologist [2]. In young women, adverse outcomes related to copper IUDs include retained devices, uterine perforation, migration, unintended pregnancy, and heavy bleeding; however, these are relatively rare in proportion to the large number of women who use copper IUDs [3]. Limited guidance exists surrounding whether it is appropriate to offer another IUD after a patient has an adverse outcome with a previous IUD. Here, we present the case of a patient who experienced multiple IUD insertions with multiple adverse outcomes.

## Case presentation

**Figure 1:**
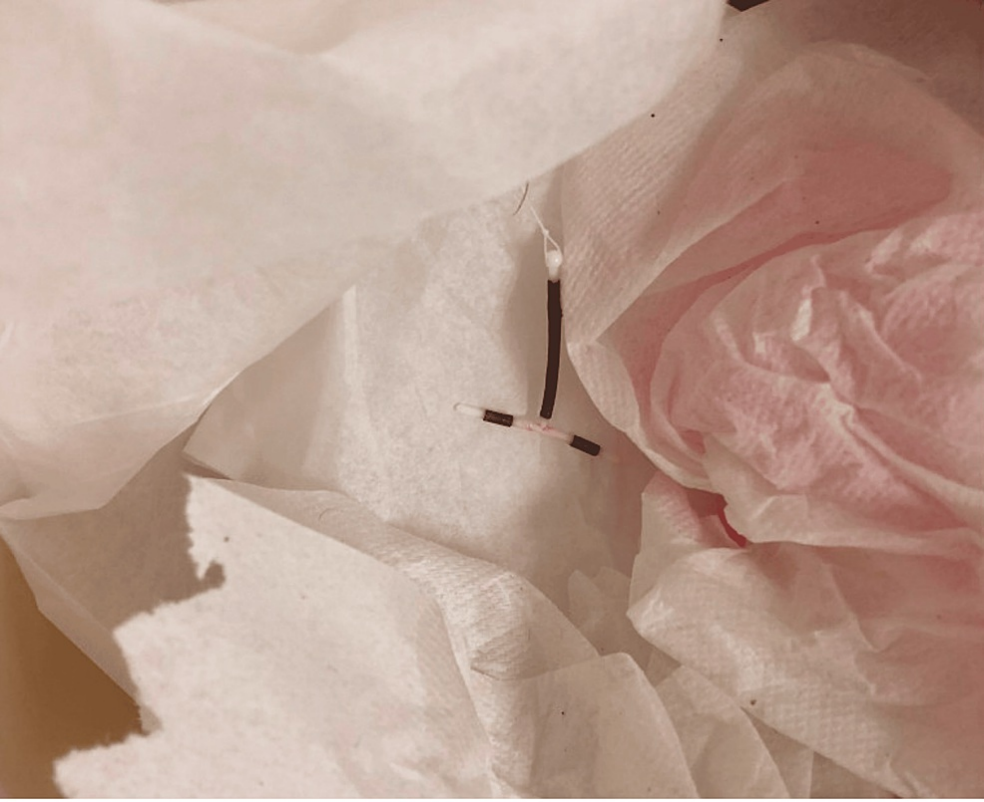
First IUD was successfully removed

CB was a female G0P0 patient in her mid-twenties who had a Paragard IUD placed by a community OB/GYN. She had no other past medical history and past surgical history and was not taking any other medications. The IUD was in place for several months until she became pregnant, which ended in elective abortion. The IUD was removed by her OB/GYN and the patient kept this device (Fig. 1).

The patient had a second Paragard IUD placed immediately after removal of the initial IUD. This IUD was in place for several months until the patient became pregnant a second time, which ended in elective abortion. She had the second IUD removed by her OB/GYN, but did not recall if she was shown the IUD upon removal.

The patient had a third Paragard IUD placed immediately after removal of the second IUD. This IUD was in place for two years with no issues.

**Figure 2:**
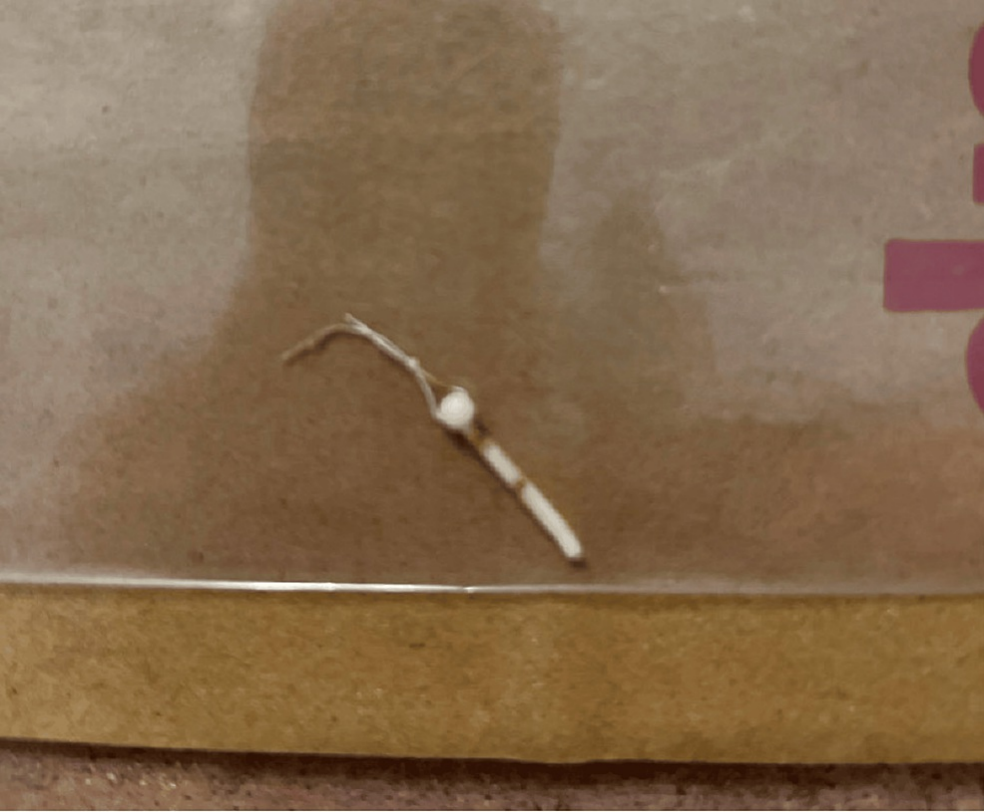
IUD fragment that had spontaneously expulsed

In 2022, at age 30, CB presented to her primary care physician in an outpatient family medicine clinic due to spontaneous expulsion of her IUD. She brought the IUD piece to the clinic (Fig. 2), which was missing two wings, had two knotted strings, and was without a copper lining. She had mild suprapubic cramping, but no vaginal discharge, no excessive bleeding, no recent trauma, and no recent sexual activity in the past week. She was menstruating at the time of presentation and had no history of STIs. Although she did not want to become pregnant at that time, she did desire fertility in the future so she did not want permanent sterilization. Upon pelvic exam, the cervix was visualized and menstrual bleeding was present. Two IUD strings were visualized in the cervical os. Based on her examination, it was determined that the piece of IUD that she had brought to the clinic that day may have been either her second or third IUD. The patient then requested to have her IUD removed as she was concerned after the piece fell out earlier in the day. Three attempts were made to remove the IUD with ring forceps, but they were unsuccessful and she was referred to a gynecologist for further management.

One week later, CB presented to a gynecologist for ultrasound-guided removal of the IUD. Upon pelvic exam, the IUD strings were visualized and one attempt was made to remove it with ring forceps, but was unsuccessful. A cervical tenaculum was placed and a second removal attempt was made with a thin grasping instrument under ultrasound guidance, but it was unsuccessful. To avoid trauma to the patient, further efforts were deferred at that time and CB was referred for surgical management.

Several months later, a hysteroscopy was performed with a second gynecologist under general anesthesia. Once the cervix was dilated and the hysteroscope was advanced, one IUD was noted to be positioned backward in the cervix. It was successfully removed using the graspers of the hysteroscope, and the uterus was noted to have a normal structure with normal ostia visualized. CB was discharged to home the same day. Following the removal of the IUD, CB had a progestin-only implant inserted in her upper arm for contraception at another facility.

## Discussion

In this case, the patient had adverse outcomes with each of her IUDs. Her first IUD failed and resulted in an unintended pregnancy. Her second IUD also resulted in failure and another unintended pregnancy. Copper IUDs have been shown to be exceptionally effective in preventing pregnancy, and according to the FDA, less than one out of every 100 patients who use copper IUDs for birth control may become pregnant. Furthermore, the efficacy of copper IUDs in preventing pregnancy has such strong evidence that they are able to be used as emergency contraception [4]. In a study by Fay et al. in 2021, no pregnancies resulted from 518 individuals who had copper or hormonal IUDs placed and had sexual activity within one week of insertion [5]. In the authors’ opinion, the first IUD failure may have been attributed to a spontaneous failure. Therefore, the authors believe that it was reasonable to offer the second IUD to the patient. However, two IUD failures resulting in pregnancy are even more unlikely than one. Although not impossible, the authors believe it would be improbable that the patient would have had two randomly defective devices. In any case, the insertion of the third IUD was unwise after two previous failures. Other alternatives for long-acting contraception, such as progestin-only implants, would have been a more suitable choice than a third IUD.

When the patient presented to clinic, she brought the first IUD that had been removed and the expulsed fragment. However, because she did not have the second IUD after removal, it was unclear whether the second IUD was the expulsed fragment or the retained device. It was also unclear whether the third IUD was the expulsed device or the retained device. Nonetheless, she experienced a spontaneous expulsion of a fractured device and a retained device in addition to her previous unintended pregnancies from failed IUDs. Multiple case reports detail how copper IUDs have fractured, become retained in various tissues, or have been unable to be located during routine surveillance [5-12]. Furthermore, a cross-sectional study by Vitale et al. in 2022 that surveyed surgeons who performed in-office IUD insertion procedures found that copper IUDs were most commonly reported to be found retained in patients [13]. While it is not uncommon for such events to occur with copper IUDs, it is highly uncommon to happen in the setting of other IUD failures. In all of the documented case reports, the patients had one adverse outcome with one IUD instead of several with multiple IUDs. The combination of adverse events highlights the importance of offering a different type of contraception once two adverse outcomes have been documented.

## Conclusions

In this case, one patient experienced multiple adverse outcomes related to multiple copper IUDs. Although there is extensive literature documenting adverse outcomes related to copper IUDs, it is highly unlikely and uncommon for one patient to have experienced so many. From this case, it is important to offer a different type of contraception to a patient who has experienced multiple failures of and adverse outcomes from IUDs. An implantable subcutaneous device (Nexplanon) was placed in order to provide contraception. At the present time, the patient has had no further complications or complaints regarding her progestin-only implant.
